# A specialized motion capture system for real-time analysis of mandibular movements using infrared cameras

**DOI:** 10.1186/1475-925X-12-17

**Published:** 2013-02-22

**Authors:** Daniel Antônio Furtado, Adriano Alves Pereira, Adriano de Oliveira Andrade, Douglas Peres Bellomo Junior, Marlete Ribeiro da Silva

**Affiliations:** 1 Laboratory of Biomedical Engineering, Faculty of Electrical Engineering, Federal University of Uberlandia, Av. Joao Naves de Avila, 2121, Uberlandia, MG CEP 38408-100, Brazil; 2 Department of Occlusion, Fixed Prosthesis and Dental Materials, Faculty of Dentistry, Federal University of Uberlandia, Av. Pará, 1720, Uberlandia, MG CEP 38400-902, Brazil

**Keywords:** Mandibular movements, Optical motion capture, Temporomandibular joint

## Abstract

**Background:**

In the last years, several methods and devices have been proposed to record the human mandibular movements, since they provide quantitative parameters that support the diagnosis and treatment of temporomandibular disorders*.* The techniques currently employed suffer from a number of drawbacks including high price, unnatural to use, lack of support for real-time analysis and mandibular movements recording as a pure rotation. In this paper, we propose a specialized optical motion capture system, which causes a minimum obstruction and can support 3D mandibular movement analysis in real-time.

**Methods:**

We used three infrared cameras together with nine reflective markers that were placed at key points of the face. Some classical techniques are suggested to conduct the camera calibration and three-dimensional reconstruction and we propose some specialized algorithms to automatically recognize our set of markers and track them along a motion capture session.

**Results:**

To test the system, we developed a prototype software and performed a clinical experiment in a group of 22 subjects. They were instructed to execute several movements for the functional evaluation of the mandible while the system was employed to record them. The acquired parameters and the reconstructed trajectories were used to confirm the typical function of temporomandibular joint in some subjects and to highlight its abnormal behavior in others.

**Conclusions:**

The proposed system is an alternative to the existing optical, mechanical, electromagnetic and ultrasonic-based methods, and intends to address some drawbacks of currently available solutions. Its main goal is to assist specialists in diagnostic and treatment of temporomandibular disorders, since simple visual inspection may not be sufficient for a precise assessment of temporomandibular joint and associated muscles.

## Background

Temporomandibular joint (TMJ) and the human mandible are part of an interesting and complex biomechanical system capable to perform several functions and high precision movements, such as chewing, swallowing and speech [1]. Systems designed to record and analyze these movements have received increasing attention in the past few years, since they provide quantitative parameters that support the clinical diagnosis and treatment of temporomandibular disorders (TMD) [2-4].

Therefore, a number of methods, techniques and devices for recording and analyzing human mandibular movements have recently been proposed [1,5-18]. Conventional mechanical methods, such as JT-3D System from BioResearch Company, usually employ an articulated mechanism fixed on the head in order to record mandibular motion. However, most of these devices just simulate a pure rotation of lower jaw along a single axis, while mandibular movements involve simultaneous rotation and translation [19].

Ultrasonic-based methods, such as JMA System of zebris Medical GmbH, typically use a face-bow together with integrated receiver sensors for acquisition of 3D mandibular movements. In this case, motion is captured by measuring the travel time of ultrasound impulses [14,20]. Similarly, some electromagnetic-based techniques, like the prototype system presented by Santos et al. (2008) [1], use a facial arc with electromagnetic sensors to record appropriate kinematics. However, these magnetic methods can be sensitive to the presence of metal in the environment [21] and their accuracy has rarely been reported.

In most cases, mechanical, electromagnetic and ultrasonic-based devices are somewhat bulky and make the patients feel unnatural during the routine tests. In addition, many of them are also relatively heavy, expensive and complicated to use [16].

Another group of methods and systems used to record jaw movements works with video cameras and passive or active markers. Some authors have presented off-line techniques employing low-cost CCD cameras [5,6,9,14,19], while others have used commercial, high-end motion capture packs [17,22,23]. By using a single CCD camera and a reflective marker fixed to the mandible, Pinheiro et al. (2011) [6] proposed a computational method for recording mandibular movements in a two-dimensional space. With a mean error of 0.4 mm, analyses were done separately in frontal and sagittal planes, but not in 3D. Further, Fang and Kuo (2008) [19] presented a system using a pair of CCD cameras and three light-emitting diodes (LED) affixed to a pair of tracking plates for 3D reconstruction. The main disadvantage of this system is the obstruction caused by the tracking plates, which can limit the subject’s freedom when executing jaw movements. The authors reported an RMS accuracy of 0.198 mm.

Commercial optical systems used by the entertainment industry to get facial expression, or even optical systems employed to capture full-body motion, could be used to register mandibular movements. Indeed, Rohrle et al. (2009) [17] and Mani et al. (2010) [23] have analyzed jaw movements using a Vicon MX system and a Qualisys five-camera system, respectively. The problem with this idea is the high cost of these systems, which usually include expensive and sophisticated software that is developed to work with dozens of markers and handle extreme conditions, like fast motion, marker occlusions and large capture volumes. However, none of these features is actually needed for tracking the mandible. Also, these systems are not specialized to perform mandibular motion capture and some actions, like marker identification or rigid body creation, may have to be done manually.

In this context, we propose a specialized optical motion capture system for mandibular movement analysis using three infrared cameras and a set of nine reflective markers. Unlike most techniques and devices recently published in literature, the proposed system brings together a set of important features, once it combines good precision and accuracy, minimum obstruction, real-time 3D reconstruction and analysis and moderate cost. Furthermore, it can give parameters of facial morphology and can automatically recognize the markers.

More specifically, our contribution includes the proposal of a reflective marker setup together with a camera set configuration, the suggestion and evaluation of some classical, already available algorithms for camera calibration and 3D reconstruction in the context of mandibular movement analyzing, and the proposal of a specialized computational method to perform automatic identification and tracking of our set of markers in real time. All these algorithms and methods are relatively simple, as they were implemented and evaluated in our prototype software. The validation test suggests a mean error of 0.156 mm and a precision of 0.259 mm within the volume intended for recording mandibular movement.

## Methods

To perform three-dimensional reconstruction of condylar movements, we propose the usage of three infrared specialized cameras and a set of reflective markers that must be arranged at key points of the subject’s face. In our experiments, we have used cameras model *OptiTrack Flex V100*, manufactured by NaturalPoint. These cameras are natively capable to find out white points in the images, which correspond to reflective objects (usually markers) in the scene. All cameras synchronously take images of the scene and reduce the image data to a set of 2D image coordinates representing the detections of the markers. Each camera is able to capture up to 100 frames per second, which is sufficiently high to guarantee detailed register of lower jaw movements. It is important to note that we have used only the three infrared cameras, which cost just a fraction of the whole motion capture system offered by the manufacturer (the complete system includes a 3D reconstruction software, calibration tools, USB hubs, etc.).

### Marker set

A set of nine retro-reflective markers is proposed to allow mandibular movement analysis. Eight of them are called the *secondary tracking markers* and their purpose includes estimating some morphological parameters of the mandible. The *primary tracking marker* is the one primarily employed to track the movement of the jawbone.

**Figure 1:**
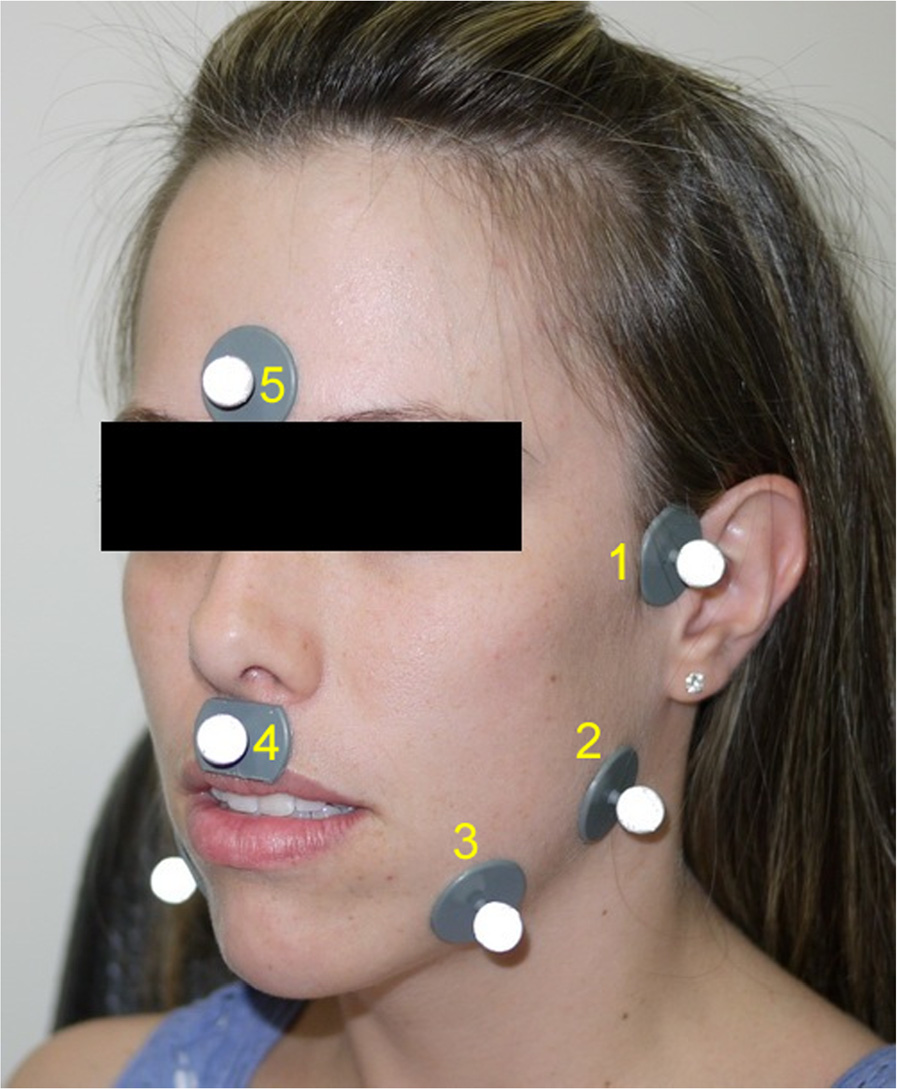
**Proposed marker setup.** (1) TMJ external surface; (2) mandible angle region; (3) middle region between the chin and the mandible angle; (4) above upper lip and (5) on the forehead. Markers in positions 1, 2 and 3 are placed on both sides of the face. Smaller ones could also be used.

Secondary tracking markers can be fixed on skin by using adhesive tape and a plastic support. They must be positioned on the following regions of the face: (1) TMJ external surface (left and right), (2) mandible angle region (left and right), (3) middle region between the chin and the mandible angle (left and right), (4) above upper lip and (5) on the forehead. Figure 1 illustrates these points.

Due to the movement of the skin over the bones, 3D coordinates of the secondary markers do not exactly correspond to the real positions of the underlying bones. However, these coordinates can be used to support the alignment of the head, to provide a visual reference for three-dimensional analysis and to give a rough approximation of the facial morphology of the subject, since facial morphology can influence condylar movements [3,24]. Therefore, mandibular parameters such as gonial angle, bigonial width and bicondylar width can be estimated from the reconstructed 3D coordinates of the secondary markers.

**Figure 2:**
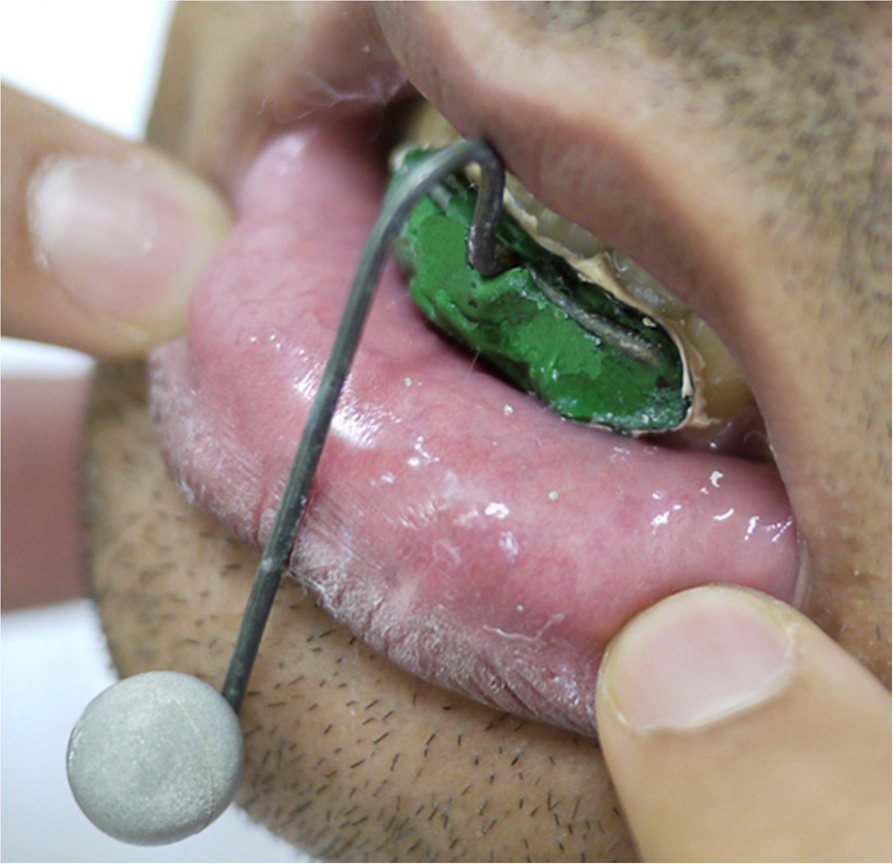
**Primary marker.** Primary tracking marker is rigidly fixed to the mandible using a metal marker support, which is attached between the inferior lip and the incisors.

To maximize accuracy and precision, the *primary tracking marker* must be set firmly to the mandible. In our experiments, we have employed a metal support fixed inside the mouth. The support was placed between the inferior lip and the labial surface of the lower incisors, as illustrated in Figure 2. A thermoplastic material (godiva) was used to make the fixing base and a zinc-enolic paste (Lyzanda®) was employed as adhesive material making the interface between teeth and the base. As a result, the path traveled by the primary marker is truly related to the path traveled by the mandibular condyle [13].

The forehead marker aims to estimate the movement of the head. In fact, mandibular movements are a combination of condylar and head movements [25]. Therefore, the movement of the incisive point can be compensated by subtracting the movement of the marker on the forehead from the movement of the primary marker [6].

### Camera configuration

**Figure 3:**
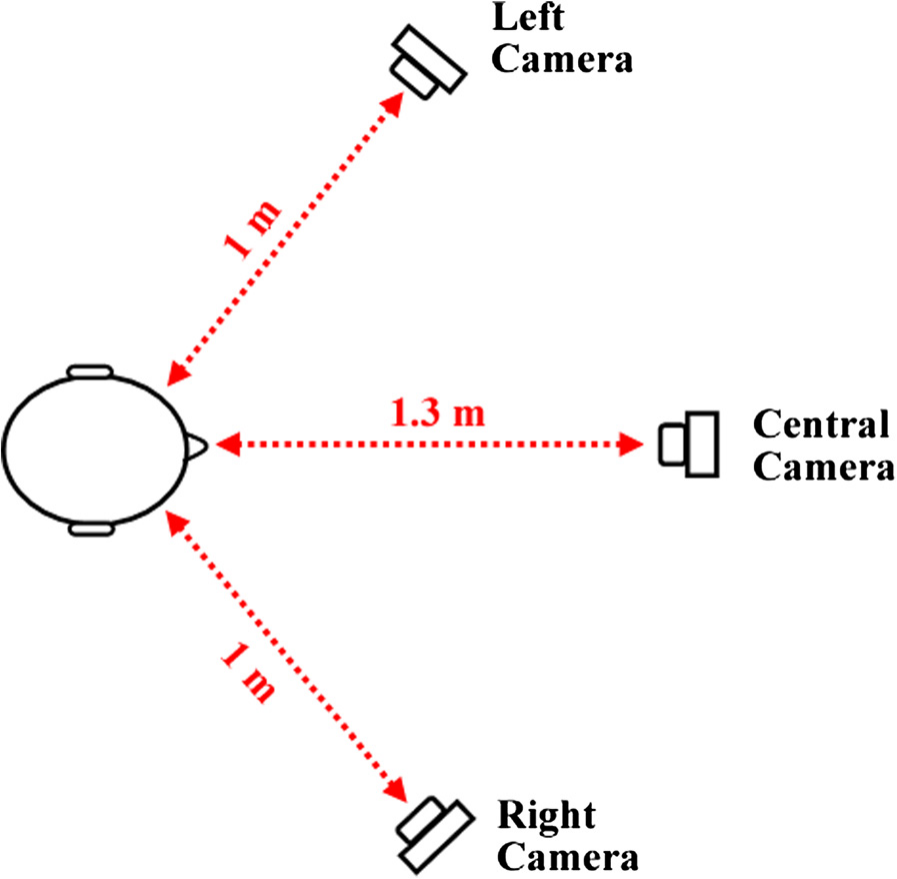
**Top view of the proposed camera setup.** The left and right cameras are placed at 1 meter distance from the subject forming an angle of 120 degrees. The central camera is positioned at 1.3 meters distance.

In order to allow 3D reconstruction, we need each marker to be seen by at least two cameras. Considering our set of markers, this condition can be satisfied by placing one camera in front of the subject (to see all markers) and one on each side (left and right). The proposed configuration is presented in Figure 3.

**Figure 4:**
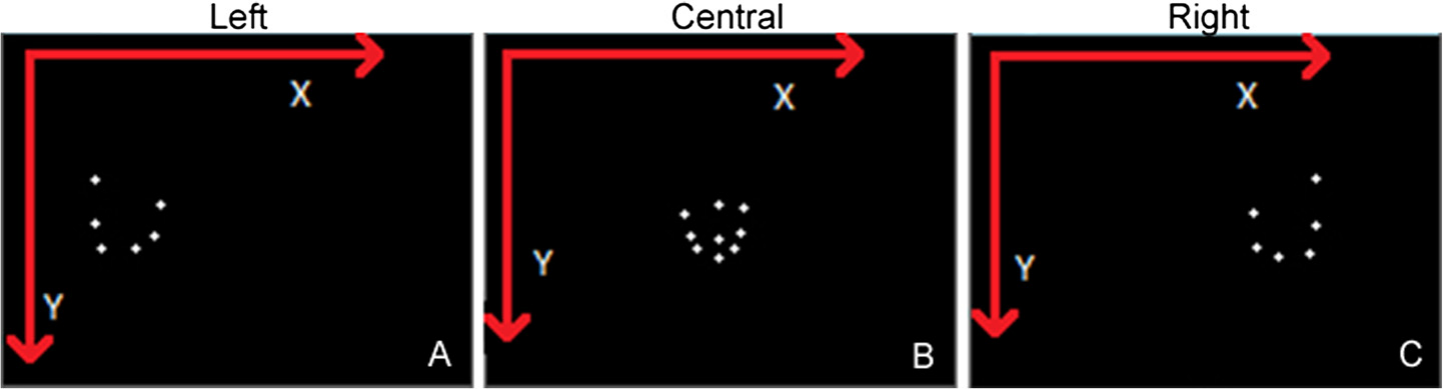
**Infrared camera detections of the markers.** The images were created using 2D data produced by the infrared cameras during a capture session. White dots represent detected points in each camera. Subject was with his mouth closed. The arrows emphasize the origin (0, 0) and the growing direction of image coordinates.

The left and right cameras must be placed at about 1 meter distance from the subject, forming an angle of approximately 120 degrees and the central camera should be located at 1.3 meters from the subject. The three cameras must be vertically positioned about 20 cm above the head line. With this camera configuration, the dental specialist can freely move himself around the subject while the shot distances are still sufficient to ensure good precision and accuracy when detecting the markers. Additionally, this arrangement enables automatic marker identification using the proposed algorithm that is presented over the next sections. Vertical position was defined above the head line in order to avoid merging of detected points, especially for the central camera. Note that the central camera can see all the nine markers, but the left and right cameras cannot see the markers on the opposite side. A sample of the detected points is illustrated in Figure 4.

### Camera calibration

Before calculating three-dimensional data, cameras need to be calibrated. Camera calibration is a widely used procedure in computer vision for extraction of metric information from the scene where the images will be taken. The process aims to find out the exact position and orientation of the camera in space, as well as the camera internal aspects, like focal length and image sensor position. All these parameters allow one to define a mathematical correspondence between the coordinates from the image plane (given in pixels) and the world space coordinates (given in some unit of length). From the several calibration techniques currently available [26,27], we decided to implement the well-known Direct Linear Transformation (DLT) approach [28], since it is relatively simple and provides good precision for 3D calculation when working with small reconstruction volumes [29].

**Figure 5:**
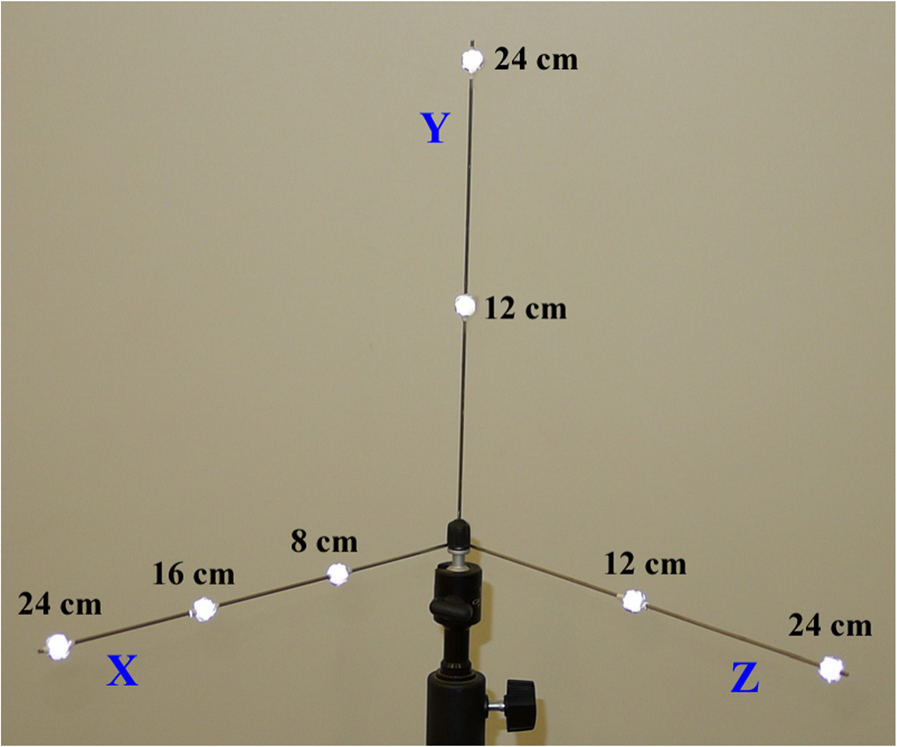
**Calibration tool designed for the system.** Three markers are used in x-axis, separated by 8 centimeters from each other, and two markers are used in each remaining y and z-axis.

DLT calibration requires the 3D coordinates of at least six points in the scene. To provide this, calibration tools keeping markers at well-known positions are commonly used. For our system, we designed a calibration tool composed by a soldered stainless steel orthogonal triad with seven attached 10 mm markers, as illustrated in Figure 5. When calibrating the system, the calibration tool must be briefly positioned (using a tripod, for example) in the region where the movements will be taken so that each camera can register at least one image of it. To maximize accuracy and precision, after calibration, mandibular movements must be executed inside the region defined by the x-y-z axes. The subject’s head must be oriented so that its Frankfort horizontal plane [30] keeps parallel to the xz plane of the triad and its sagittal plane keeps parallel to the plane defined by the y and z axes. Real-time computed 3D coordinates for the secondary markers on TMJ region can be used to support this head alignment by suggesting an eventual correction in its rotation or tilt. Such head positioning should be accomplished by a dental specialist.

### Point identification, tracking and 3D reconstruction

The proposed computational method for automatic recognition of image points and calculation of spatial trajectories of the markers can be organized in three main stages: (1) 2D point identification and inventory, (2) 2D point tracking and (3) 3D reconstruction. These stages are now discussed.

#### 2D Point identification and inventory

Before calculating 3D coordinates of the markers, detected points in different images must first be matched. That means we need to find out which points have been generated by the same reflective marker*.* Groups of 2D points satisfying this condition become ready to be used in 3D computation*.* In fact, the properties and constraints of epipolar geometry [31] could be used to solve this point correspondence problem. However, the point identification itself would remain unsolved. Instead, we propose two simple and direct algorithms to solve both correspondence and point identification problems by analyzing the image pattern of each camera (as illustrated in Figure 4, it is expected a specific pattern for each camera). The algorithm for the *central* camera is presented below:

a. Sort the image points by their x-coordinates in order to organize them from left to right. By analyzing the ordered points, we can identify three groups: (1) three points in the right side of the face; (2) three points in the middle region (forehead, upper jaw and chin) and (3) three points in the left side;

b. Sort the three points with the lowest x-coordinates (group 1) by their y-coordinates and labels the resulting points, respectively, as TMJ_RIGHT, ANGLE_RIGHT and MID_JAW_RIGHT;

c. Sort the three points with the highest x-coordinates (group 3) by their y-coordinates. The ordered points must be labeled, respectively, as TMJ_LEFT, ANGLE_LEFT and MID_JAW_LEFT;

d. Sort the three intermediate points (group 2) by their y-coordinates. Labels the ordered points, respectively, as FOREHEAD, UPPER_JAW and CHIN.

The algorithms for the left and right cameras are analogous. Thus, we described just one of them (left camera):

a. Sort the points by their x-coordinates in order to organize them from left to right. By analyzing the ordered points, we can identify two groups: (1) the three left-most points, which match the markers in middle region of the face (forehead, upper jaw and chin) and (2) the remaining three points in the left side of the face.

b. Sort the three points with the lowest x-coordinates by their y-coordinates. These points correspond, respectively, to FOREHEAD, UPPER_JAW and CHIN;

c. Sort the three points with the highest x-coordinates by their y-coordinates. The first point in the resulting list must be labeled as TMJ _LEFT;

d. Sort the last two points without label by their x-coordinates. The first ordered point must be labeled as MID_JAW_LEFT and the second, as ANGLE_LEFT.

Although the point identification algorithms previously described are able to recognize all expected 2D points, the method itself is a bit time consuming since it involves several sorting operations. Hence, we propose the execution of these algorithms for only the two initial frames of each capture session. For the following frames, however, the point identification can be performed more efficiently by means of 2D point tracking.

#### 2D Point tracking

For the next camera frames, it is possible to accelerate the point recognition by looking at the neighborhood of each 2D point identified in previous frames. As the subject is not supposed to do large range movements with the head or condyles, neither big changes in 2D detections nor occlusion of any marker during the capture session are expected. In fact, the tracking technique we suggest is a simplification of that presented by Herda et al. (2001) [32]. It assumes that the displacement of a 2D point from one frame to the next is very small, which allows us to estimate its coordinates in current frame from its coordinates in the past ones.

**Figure 6:**
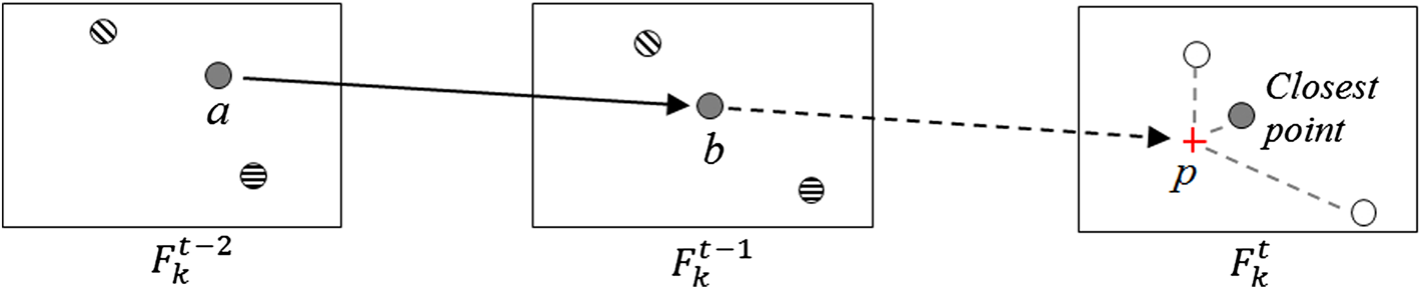
**Illustration of 2D point tracking algorithm.** The gray point in$F_{k}^{t-1}$ represents the marker being tracked. The predicted position *p* is calculated from the last positions *a* and *b.* The technique is a simplification of that presented by Herda et al. [32], since no marker occlusion or merge is expected.

Considering that $F_{k}^{t}$ stands for a frame captured at instant *t* from camera *k,* the displacement of a point from $F_{k}^{t-2}$ into $F_{k}^{t-1}$ can be used to get the point’s direction and project its expected position *p* in $F_{k}^{t}$. Once we have this predicted position, we must find the point that is closest to *p.* This point is assumed to be a detection of the same marker and must receive the corresponding label. This is illustrated in Figure 6.

Equations 1 and 2 show the direct calculation of the predicted position *p.* Terms *a* and *b* represent, respectively, the coordinates of the tracking point in $F_{k}^{t-2}$ and $F_{k}^{t-1}$.

(1) $$p_{x}=b_{x}+\left(b_{x}-a_{x}\right)$$

(2) $$p_{y}=b_{y}+\left(b_{y}-a_{y}\right)$$

#### 3D Marker reconstruction

Three-dimensional reconstruction is computed based on the DLT method. As soon as 2D points become identified, those with the same identification (same label) must be used to triangulate the 3D coordinates of the corresponding physical markers. As the markers on the left side of the face (TMJ, angle and mid jaw) are seen by only the left and central cameras, the pair of 2D points for each of these markers is employed for composing a DLT-based overdetermined linear system whose solution will lead to the related spatial coordinates.

This system can be expressed as the matrix multiplication of the Equation 3. $L_{i}^{l}$ and $L_{i}^{c}$ stand for the i-th DLT coefficient of the left and central cameras, respectively, and *u*^*l*^ and *v*^*l*^ (resp. *u*^*c*^*v*^*c*^*)* are the coordinates of a point in the image captured by the left camera (resp. central). An exact solution for this system is not possible, but we can find a solution that minimizes the residual error by using the SVD technique [33].

(3) $$\left[\begin{array}{l} \left(L_{1}^{l}-u^{l}L_{9}^{l}\right)\;\left(L_{2}^{l}-u^{l}L_{10}^{l}\right)\;\left(L_{3}^{l}-u^{l}L_{11}^{l}\right)\\ \left(L_{5}^{l}-v^{l}L_{9}^{l}\right)\;\left(L_{6}^{l}-v^{l}L_{10}^{l}\right)\;\left(L_{7}^{l}-v^{l}L_{11}^{l}\right)\\ \left(L_{1}^{c}-u^{c}L_{9}^{c}\right)\;\left(L_{2}^{c}-u^{c}L_{10}^{c}\right)\;\left(L_{3}^{c}-u^{c}L_{11}^{c}\right)\\ \left(L_{5}^{c}-v^{c}L_{9}^{c}\right)\;\left(L_{6}^{c}-v^{c}L_{10}^{c}\right)\;\left(L_{7}^{c}-v^{c}L_{11}^{c}\right) \end{array}\right]\;\left[\begin{array}{l} x\\ y\\ z \end{array}\right]\;=\;\left[\begin{array}{l} u^{l}-L_{4}^{l}\\ v^{l}-L_{8}^{l}\\ u^{c}-L_{4}^{c}\\ v^{c}-L_{8}^{c} \end{array}\right]$$

For the right markers, the same process can be applied and for the central markers (forehead, upper jaw and chin), 3D reconstruction is achieved using the three detections of each marker to mount a similar linear system with six linear equations. Note that point identification, tracking and 3D reconstruction process is able to return the spatial coordinates of the nine considered markers for each instant *t*. So, the desired 3D trajectories through time can be generated directly from these reconstructed points. A detailed description about using DLT to compute 3D coordinates can be found in [34].

It is important to mention that during a motion capture session, the subject must keep the head as immobile as possible. In our experiments, this requirement was easily met by using a high-density foam support touching the occipital region of head and neck for head partial immobilization. All participants of these experiments provided written informed consent to participate and the study was approved by the Ethical Committee of Federal University of Uberlandia, Brazil (process number 010/10).

### 3D Data Pre-processing

3D trajectory of the primary marker can be smoothed using a digital Butterworth filter with 4 poles and a cut-off frequency of 8 Hz. According to Miles (2007) [35], the mandible voluntary movements together with tremors can reach a frequency of 6–7 Hz. Therefore, noise components with frequency greater than 8 Hz are attenuated, giving a smoother appearance to the movement trace.

### Software implementation aspects

A prototype software was implemented to communicate with the cameras and perform all the tasks previously described, including camera calibration, 2D point match, marker identification, tracking and 3D reconstruction. The software was programmed using the Microsoft .NET Framework with C# language and all 3D objects and trajectories were rendered in real-time using the Microsoft DirectX SDK. We have used Intel’s OpenCV library to solve the overdetermined linear systems and the experiments were performed in a desktop computer powered by a quad-core 2.8 GHz Intel processor with 4 GB of RAM.

## Results

### System validation and reliability

**Figure 7:**
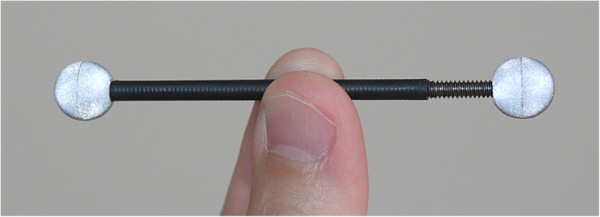
Rigid bar of 70 mm used to evaluate accuracy and precision.

The validity and reliability of motion analysis systems have been evaluated in terms of precision, accuracy and repeatability [36,37]*.* In order to estimate the accuracy and precision of the proposed system, we performed a classical experiment [6,38-40] by using a reference rigid bar of 70 mm length with two markers attached to its extremities (we implemented a software procedure to track the 2D points and reconstruct the corresponding 3D coordinates). The bar was moved randomly through the measurement volume during 30 seconds and the distance between the markers was calculated for each frame. The estimated distance values were then compared to the known (true) value of 70 mm. The accuracy was evaluated by determining the root-mean-square (RMS) error associated to the measured distances and the precision was estimated by computing the standard deviation of those distances, the maximum distance error and the 95% confidence interval. The calculated values are presented in Table 1 and the employed bar is illustrated in Figure 7.

**Table 1:** Results of the system validation test

**True 3D distance (mm)**	**Estimated (mean) 3D distance**	**RMS accuracy (mm)**	**Standard deviation (mm)**	**Minimum distance (mm)**	**Maximum distance (mm)**	**95% CI (mm)**
70.000	70.156	0.259	0.208	69.501	70.683	0.481

A reference rigid bar of 70 mm length with two markers was moved through the reconstruction volume during 30 seconds and the 3D distance between the markers was estimated for each image frame.

Test-retest reliability was quantified by performing the rigid bar test three more times at intervals of one week. Right before starting each test session, cameras were set-up according to the distances and positions we have proposed. The calibration procedure was conducted every time and after each session the system was disassembled again. For the computed bar length, analysis of variance (ANOVA) test revealed no significant differences across the tests (p > 0.24). The standard deviation of the measurements was less than 0.2 mm for all sessions and the distance mean error was always less than 0.16 mm.

### Clinical experiments

To investigate the clinical application of the system, it was used to evaluate mandibular movements in a group of 22 subjects. According to Research Diagnostic Criteria for Temporomandibular Disorders (RDC/TMD) questionaire, 20 of these subjects presented no signs or symptoms of TMD and two subjects evidenced masticatory dysfunction.

When starting the capture, the subjects were instructed to sit down in a long back chair with the head supported by a rigid foam. The goal was to help them sense the head position from proprioceptive stimulus and avoid head displacement while executing mandibular movements. They were oriented to execute three types of jaw movements: (1) maximum opening-closing, (2) maximum lateral excursions (left and right) and (3) maximum protrusion, which are all specific movements for the functional evaluation of the mandible. In order to acquire the average parameters, the subjects were oriented to repeat each type of movement 6 times, resulting in 24 cycles.

**Figure 8:**
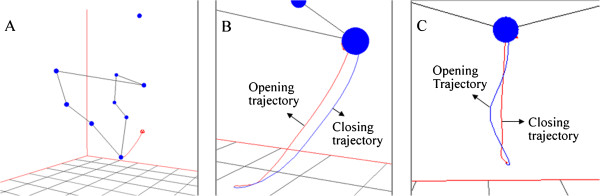
**Model 3D and trajectories reconstructed by the prototype software.** The images correspond to an opening-closing jaw movement executed by a subject without symptoms of TMJ disorders. (**A**) Mouth in maximum aperture. The red line represents the primary marker opening trajectory. (**B**) Perspective zoomed view of 3D opening-closing trajectory. (**C**) Frontal view zoomed in trajectory.

For all subjects, the system was able to automatically detect the markers, track them and reconstruct their 3D coordinates frame by frame. The corresponding 3D model was always available for real-time visualization and analysis. Figure 8 shows the reconstructed markers and the trajectory travelled by the primary marker during an opening-closing movement performed by a subject without symptoms of temporomandibular dysfunctions. Blue points correspond to spherical markers. Figure 8(A) shows the markers when the mouth was in maximum aperture. The opening trajectory defined by the primary marker is presented in red line and the complete trajectory is presented from different angles and zooms in Figure 8(B) and Figure 8(C).

**Figure 9:**
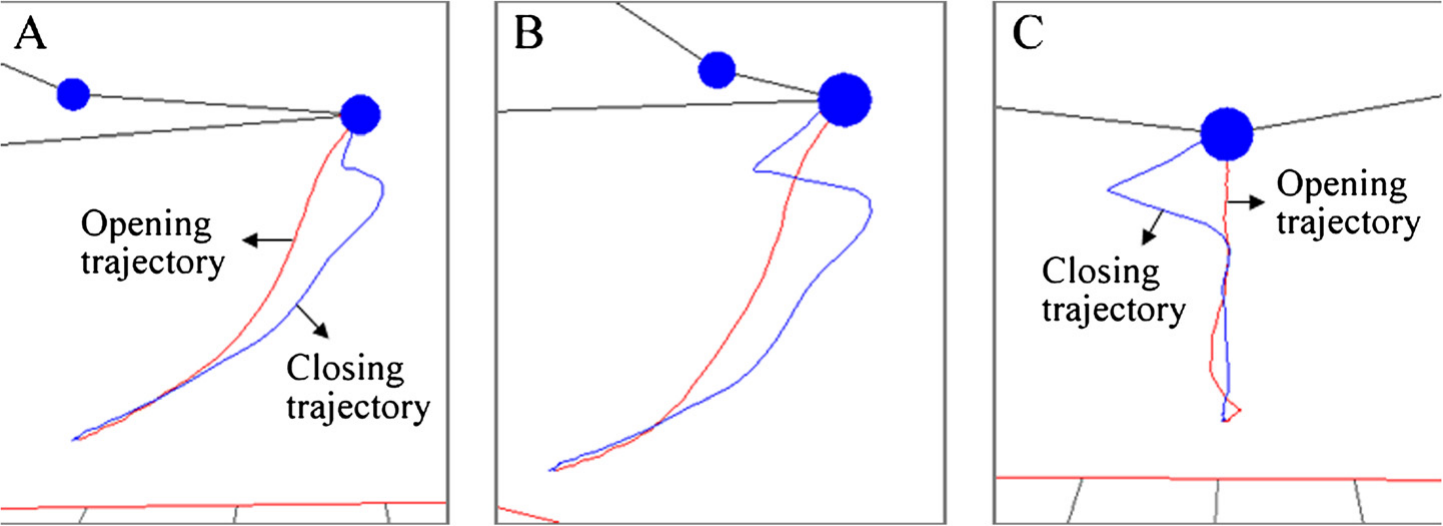
**Trajectory 3D of the primary marker.** Movement registered along an opening-closing jaw movement performed by a subject with masticatory dysfunction. Trajectory is shown from three different angles: (**A**) lateral view, (**B**) perspective view and (**C**) frontal view.

Figure 9 shows the trajectory of the primary marker along an opening-closing jaw movement executed by a subject with symptoms of TMD. The 3D trajectory is presented from lateral (Figure 9(A)), perspective (Figure 9(B)) and frontal (Figure 9(C)) views. The closing trajectory provides evidence for the abnormal function of the temporomandibular joint, since the lower jaw suddenly goes out of the expected track when finishing the movement cycle.

Table 2 presents the mean values of the maximum displacement registered by the system in each type of jaw movement. Means were calculated for the 20 healthy subjects, where OC-VMax stands for **v**ertical (y-axis) displacement in the **max**imum aperture of the mandible during **o**pening-**c**losing movement and OC-DMax stands for **d**epth (z-axis) displacement in the **max**imum aperture of the same movement. Similarly, LL-HMax stands for horizontal (x-axis) displacement in the **max**imum **l**eft **l**ateral excursion. RL and PR stand for **r**ight **l**ateral and **pr**otrusion, respectively. A dentistry specialist selected the start, the maximum, and the end points for each cycle of movement in the program.

**Table 2:** The means of the maximum displacement of the primary marker

**Movement**	**Mean Value (mm)**
OC-**H**Max	1.31 ± 0.61
OC-**V**Max	39.04 ± 7.69
OC-**D**Max	27.76 ± 9.60
LL-**H**Max	9.50 ± 3.13
LL-**V**Max	3.73 ± 1.18
LL-**D**Max	1.97 ± 1.29
RL-**H**Max	9.56 ± 3.42
RL-**V**Max	3.41 ± 1.26
RL-**D**Max	1.56 ± 1.37
PR-**H**Max	1.47 ± 1.66
PR-**V**Max	3.63 ± 2.17
PR-**D**Max	7.29 ± 2.45

The values were calculated in the axes x (horizontal), y (vertical) and z (depth) for each type of movement for the functional evaluation of the mandible.

## Discussion

The registered jaw movements for the 20 healthy subjects were considered normal, with trajectories and parameters according to expected patterns and ranges [3,8,22]. For the subject with symptoms of TMD whose data were previously presented, the reconstructed 3D trajectory revealed the severity of the disorder and the exact behavior of the temporomandibular joint when the mandible went out of the expected path. These data can be used by the specialist for a better clinical assessment and for monitoring the evolution of the proposed treatment.

As presented before, the primary marker fixing technique guarantees a precise tracking of the incisal point. Anyway, this is somewhat invasive method, which can cause a minimal discomfort to the subject and consume a bit of time during the fixing process. Depending on the type of movement being captured and the purpose of the experiment, a small displacement error could be tolerated by putting the primary marker easily and directly over the chin, without the metallic support. According to Dworkin and LeResche (1992) [41], for example, lateral displacement of chin and lower jaw is of similar magnitude, as well as the chewing rhythm, which make the over skin marker a possibility when capturing these parameters. This non-invasive measuring system would enable a patient perform a masticatory movement under almost natural conditions [14].

As presented in Table 1, when validating the system we found an RMS accuracy of 0.259 mm and a precision of 0.208 mm within the volume defined by the calibration triad. Although this accuracy value is higher than the 0.198 mm reported by Fang and Kuo (2008) for their device [19] and greater than those published for the JMA ultrasonic system [20,42], the proposed method has the advantage of being much less obtrusive than these systems. When compared to other electromagnetic and optical techniques [8,14,43], including the previous 2D optical system described by Pinheiro et al. (2011) [6], the proposed system has presented similar or better trueness and precision with the additional capability of supporting real-time assessment. Furthermore, the need to perform the calibration procedure for each new capture session in [6] does not occur in the current proposal. In addition, low maximum error values acquired during the validation test (see Table 1), as well as the 95% confidence interval for the distance error confirm the reliability and robustness of the proposed system. Table 3 summarizes the properties of some of the previously mentioned techniques and commercial systems in comparison to our method.

**Table 3:** A comparison between the proposed system and some recently published techniques and commercial systems

	**3D analysis**	**Real-time**	**Cost**	**Mean error (mm)**	**RMS error (mm)**	**Overall precision (mm)**	**Mandibular morphology estimation**	**Obstruction**	**Specialized for mandibular analyzing**
**Proposed System**	yes	yes	moderate	0.156	0.259	0.208	yes	low	yes
Optical by Pinheiro et al. (2011) [6]	no	no	low	0.400	-	0.300	no	low	yes
Optical by Fang and Kuo (2008) [19]	yes	-	moderate	0.177	0.198	0.096	no	high	yes
Optical-CT by Koseki et al. (2007) [14]	yes	no	high	0.200	-	-	yes	low	yes
Magnetic by Santos et al. (2008) [1]	yes	no	low	-	-	-	-	high	yes
Magnetic by Yoon et al. (2006) [8]	yes	no	moderate	0.320	-	0.600	no	moderate	no
Ultrasound JMA [20,42]	yes	yes	moderate	~0.1	-	< 0.1	no	high	yes
Commercial high-end mocap systems	yes	yes	high	< 0.1	< 0.1	< 0.1	yes	low	no

## Conclusions

In this paper, we presented a specialized optical motion capture system for analysis of human mandibular movements using three infrared cameras and a set of nine reflective markers. The proposed system can be made with a moderate cost and provides real-time 3D reconstruction and analysis. Furthermore, it causes a minimum obstruction, can give parameters of facial morphology and can automatically recognize the markers.

To evaluate the algorithms and computational methods, we developed a prototype software and the entire system was employed to reconstruct several movements for the functional evaluation of the mandible in a group of 22 subjects. The system was able to automatically recognize the markers, reconstruct their spatial coordinates and track them in all sessions. The spatial trajectories computed for the incisal point were effectively used to confirm the normal function of TMJ in 20 subjects and to highlight the abnormal behavior of temporomandibular joint in the remaining two. Measured displacements in sagittal, frontal and horizontal planes for each type of movement were in agreement with expected ranges. The measured RMS accuracy of the system was 0.259 mm.

The proposed system is an alternative to the existing optical, mechanical, electromagnetic and ultrasonic-based methods. By using specialized infrared cameras, the system benefits from high accurate marker detection and good capture frequency. At the same time, sophisticated and expensive commercial motion capture software may be dispensed, considering that the proposed computational methods and algorithms are relatively simple and can be implemented with reduced cost. The main goal of the system is to assist specialists in diagnostic and treatment of temporomandibular disorders, since simple visual inspection is subjective and may not be sufficient for a precise assessment of temporomandibular joint and associated muscles.

## Abbreviations

TMJ: Temporomandibular Joint; TMD: Temporomandibular Disorder; DLT: Direct Linear Transformation; SVD: Singular Value Decomposition; OC: Opening-Closing; LL: Left Lateral excursion; RL: Right Lateral excursion; PR: Protrusion; VMax: Maximum vertical displacement; HMax: Maximum horizontal displacement; DMax: Maximum depth.

## Competing interests

The authors declare that they have no competing interests.

## Authors’ contributions

All authors participated in the research design, data analysis and data interpretation. DAF also participated in algorithms developing, software implementation and manuscript writing. AAP also participated in system design, data collection and manuscript revision. AOA also participated in data collection and manuscript revision. DPBJ also participated in calibration tool construction, data collection and manuscript revision. All authors read and approved the final manuscript.
